# Dabrafenib and trametinib activity in a patient with BRAF V600E mutated and microsatellite instability high (MSI-H) metastatic endometrial cancer

**DOI:** 10.1186/s40164-016-0061-2

**Published:** 2017-01-10

**Authors:** Michele Moschetta, Gabriel Mak, Joana Hauser, Catriona Davies, Mario Uccello, Hendrik-Tobias Arkenau

**Affiliations:** 1 Drug Development Unit, Sarah Cannon Research Institute, 93 Harley Street, London, W1G 6AD UK; 2 University College London, London, UK

**Keywords:** Dabrafenib, Trametinib, BRAF V600E mutation, Microsatellite instability, Endometrial cancer

## Abstract

**Background:**

Targeting BRAF V600E mutation has been proven effective in the treatment of several types of cancer. In endometrial adenocarcinoma, the BRAF V600E mutation has been rarely reported. Whether targeting BRAF oncogene may represent a plausible therapeutic strategy for the rare patients with BRAF-mutated endometrial cancer remains to be ascertained in prospective studies.

**Case presentation:**

We report herein the case of a heavily pre-treated patient with recurrent microsatellite instability high (MSI-H) BRAF V600E mutated endometrial adenocarcinoma, which was successfully treated with the V600E targeting agent dabrafenib. After developing resistance to this agent, the MEK targeting agent trametinib was added to dabrafenib achieving again a therapeutic response.

**Conclusions:**

This case shows that dabrafenib both as monotherapy and when combined with trametinib may exert significant therapeutic activity in heavily pretreated BRAF V600E mutated endometrial adenocarcinoma, and highlight potential benefits of personalized treatment in this disease.

## Background

Endometrial cancer is the most frequent gynecological tumor in developed countries, and its incidence is increasing [1]. Most patients are diagnosed with early stage disease confined to the uterus, and treated with combination of surgery and adjuvant chemotherapy depending on histology and stage [1]. Metastatic disease is however characterized by a dismal prognosis due to limited therapeutic options mainly based on platinum and taxane chemotherapy [2, 3].

The advent of sequencing technologies allowing for rapid, low cost, and accurate sequencing of clinical samples, has led to a different approach, namely personalized therapy, to the treatment of cancer based on target therapies; whether this approach increases survival compared to the conventional chemotherapy-based one remains to be ascertained [4].

Of note, the therapeutic activity of tumor-targeted agents depends not only on the presence of a specific targetable mutation, but also on the specific tumor type that harbors the mutation.

For example, agents targeting the BRAF V600E mutation can lead to impressive response rate in BRAF V600E mutated melanoma [5] and hairy cell leukemia [6], alone or when combined with a MEK targeting agent [7]; intermediate activity has been observed in BRAF mutated non-small cell lung cancer [8] and thyroid cancer [9], while minimal activity has been achieved in BRAF V600E mutated colorectal cancers [10].

BRAF mutations have been found in 15% of all cancer with BRAF V600E mutation being the most well characterized mutation leading to dysregulated BRAF activation [11].

In endometrial cancer, the frequency of BRAF mutations have been reported as being very low and occurring with a frequency of 2–5% depending on the specific case series [12]. Incidence of BRAF V600E mutation is estimated as 0.1% in the largest case series reported so far [12]. Contrary to what has been described in colorectal cancer, no association with microsatellite (MSI) status has been found [13].

Whether BRAF targeting agents may exert therapeutic activity in BRAF V600E mutated endometrial cancer remains to be determined.

To the best of our knowledge, here we provide first evidence that a BRAF targeting agent has therapeutic activity in endometrial cancer harboring a BRAF V600E mutation both alone and when combined with a MEK inhibitor.

## Case report

In 2001, a 49-year-old patient underwent total abdominal hysterectomy and bilateral salpingo-oophorectomy for a stage FIGO 1b well differentiated adenocarcinoma of the endometrium with early myometrial invasion, no adjuvant therapy was indicated. Eleven years after the initial diagnosis a recurrence in the left pelvic sidewall was identified by PET–CT imaging. She received 6 cycle of carboplatin/paclitaxel chemotherapy followed by radical radiotherapy with good partial response, and a small residual soft tissue remaining in the left pelvic side wall.

Two years later a CT scan showed significant increase in size of the pelvic soft tissue mass and she was treated with letrozole based on a 30% estrogen receptor positivity identified on histopathological review of the original tumor tissue, but rapidly progressed after 2 months of treatment. The tumor tissue was further tested for MSI status and BRAF mutational status and found to be MSI-high and to harbor a BRAF V600E mutation.

Immunohistochemistry for MLH1, PMS2, MSH2, and MSH6 proteins was first used to evaluate microsatellite status but results were inconclusive due to poor fixation of the tissue. Microsatellite status was then investigated by PCR with 4 out of 5 microsatellite markers analyzed (BAT-25, BAT-26, MONO-27, NR-21, NR-24) showing evidence of instability confirming that the tumor was indeed MSI-high. BRAF mutational status was evaluated by standard PCR-based approach.

Given the patient’s cancer family history notable for her father and aunt developing colon cancer, her mother developing pancreatic cancer and her sister developing breast cancer—BRCA 1/2 mutational testing, as well as Lynch syndrome germline mutation testing were undertaken but no mutations were identified.

She then received second line chemotherapy with gemcitabine and carboplatin completing 6 cycles; an end of treatment CT scan showed disease progression with development of new bilateral lung metastases.

**Fig. 1:**
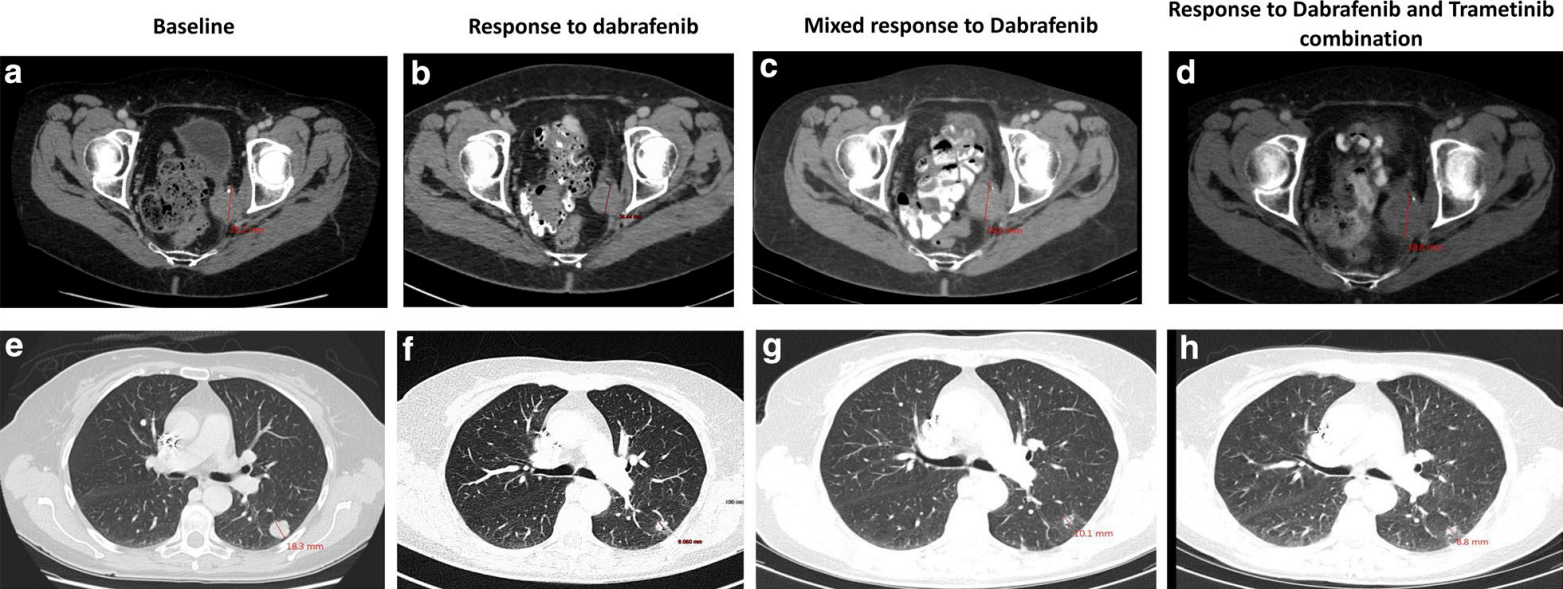
Representative CT scan images of target lesions showing response to dabrafenib monotherapy and dabrafenib and trametinib combination. Patient started treatment and showed response at the first 3 monthly CT scan (**b**, **f**) in all target lesions compared to the baseline (**a**, **e**). At the second 3 monthly CT scan the pelvic mass showed increased size (*panel* **c**, indicated by *straight red line*) compared to baseline while pulmonary metastases showed maintained response (*panel* **g**, representative image). Introduction of trametinib halted further progression of the pelvic mass (*panel* **d**) and induced further regression of the pulmonary metastases (*panel* **h**) suggesting that MEK inhibition can synergize and revert resistance to BRAF targeting agents in endometrial cancer harboring BRAF V600F mutation

The patient was subsequently enrolled in a phase 1 clinical trial (NCT02223247) and treated with a combination of weekly paclitaxel and a fatty acid synthase inhibitor. A gradual interval increase in the size of the lung metastases and the left pelvic sidewall mass was observed on 8 weekly CT scans, meeting the criteria of disease progression by RECIST 1.1 after 5 cycles of treatment (Fig. 1a, e).

As she had evidence of BRAF V600E mutation, she was then enrolled on another clinical trial (a phase 1 Pharmacokinetics Study of the Effects Rabeprazole and Rifampin on Dabrafenib in Subjects with BRAF V600 Mutation Positive Tumors, NCT01954043) and commenced treatment with therapeutic dose of dabrafenib 150 mg BD in combination with rabeprazole and rifampin for only the first cycle, tolerating treatment with few side effects. A CT scan performed after the initial 3 months of treatment showed reduction in size of all the lung metastases and the left pelvic soft tissue mass (Fig. 1b, f). Subsequent CT scan performed after an additional 3 months of dabafenib monotherapy showed continued response of the lung metastases (Fig. 1g), but slight increase of the size of the pelvic mass (Fig. 1c). Thus, 6 months after commencing dabrafenib, trametinib 2 mg therapeutic dose was commenced in combination with dabrafenib within a named patient access program. A re-staging CT scan performed after 3 months of combination treatment showing maintained response of the lung metastases and stable size of pelvic mass (Fig. 1d, h). At the time of reporting, the patient remains on treatment without experiencing any side effects and with maintained response.

## Discussion

To the best of our knowledge, this is the first report showing clinical utility of dabrafenib, alone or combined with trametinib, in a patient with BRAF V600E mutated endometrial adenocarcinoma. Among gynecological cancers, activating BRAF mutations have been more frequently found in low grade ovarian cancers, but are extremely rare in other histological types of ovarian cancers, in cervical carcinomas, and in endometrial carcinomas [12]. In endometrial carcinomas, most of the series have shown a very low prevalence of BRAF mutations with BRAF V600E mutation being very rarely found [13].

The present report highlights the importance of molecular characterization of sporadic cancers that can unexpectedly open new therapeutic options for patients. Precision medicine refers to therapeutic decisions guided by the molecular or genomic features of a tumour rather than on the basis of clinicopathological features; this novel approach has successfully been applied in this case with significant benefit for the patient; in fact, progression after the third line of chemotherapy would have left this patient without further conventional therapeutic options. However, with the revelation of an actionable mutation, this patient received a BRAF targeting agent with subsequent benefit. Although the benefits of precision medicine and personalized treatment in oncology have been recently put into question by the SHIVA trial [4], this case illustrates that at least for some patients personalized treatment may significantly impact on survival and prognosis. In this context, results from the ongoing MOSCATO trial (NCT01566019) are largely awaited. In order to maximize benefits of personalized treatment, future prospective studies need to be better designed through better patients’ selection based on strong and reliable biomarkers, and new potent and selective inhibitors should be developed.

The presence of an activating BRAF V600E mutation associates with microsatellite instability in sporadic colon-cancer tumors but not in those secondary to Lynch syndrome [14] and is mostly due to hypermethylation of MLH1 gene promoter [14]. In endometrial cancer a MSI high status has been reported in 15% of unselected endometrial cancer cases [15]; however, an absence of correlation between MSI status and BRAF V600E mutation has been reported [13], although giving the extreme rarity of the mutation in this disease, this association would be hard to be demonstrated.

Interestingly, this patient’s tumor was found to be both MSI high and to carry an activating mutation of the BRAF gene. Of note, genetic screening for Lynch syndrome was negative, suggesting, at least for this case, that the MSI and BRAF mutational status may correlate in sporadic but not in Lynch syndrome associated endometrial cancers, similarly to what described for colorectal cancers.

Of importance, MSI high tumors are known to have a high mutation load, which has been correlated with increased PD-1 expression and higher T-cytotoxic cell infiltration [16]. Data from a recently published phase 2 trial of pembrolizumab, an anti-PD-1 inhibitor, supports the hypothesis MSI high tumors, including endometrial cancers, are highly responsive to immune checkpoint blockade. Of note in this study, 1 complete response and 1 partial response were observed among the two endometrial cancer patients enrolled [17]. This preliminary clinical evidences suggest that this patient will possibly benefit by an anti-PD-1/PD-L1 agent in the future, further demonstrating the importance of molecular characterization of sporadic cancers.

After developing partial resistance to dabrafenib this patient received a MEK inhibitor in addition to dabrafenib, which at least in melanoma patients, has shown to both reverse resistance to BRAF target agents and to significantly prolong survival compared to BRAF targeting agent monotherapy [7].

In line with this evidence, the addition of the MEK inhibitor trametinib lead to resensitization of the tumor to the upstream inhibition of BRAF oncogene, thus further prolonging response to BRAF inhibitor based treatment and suggesting that combination of target treatments can be a better therapeutic strategy for this type of disease. This hypothesis is supported by the evidence that selumetinib, another selective, orally-available, MEK inhibitor [18], has shown minimal single agent activity in a phase II, single-arm, open-label study conducted in 54 recurrent EC patients [19]. However, an ongoing randomized trial in endometrial cancer patients (GOG-229O) is currently investigating the activity of trametinib versus the combined MEK/AKT inhibition with GSK2141795 and trametinib (NCT01935973) and will provide further evidences supporting this hypothesis.

## Conclusions

This case illustrates the importance of molecular characterization of sporadic cancers and the potential impact that personalized therapy can have on the prognosis of cancer bearing actionable mutations. Importantly, this case shows that MAPK pathway plays a central role in the pathogenesis of a subset of endometrial cancers and that BRAF and MEK inhibitors may exert significant anti-tumor activity in BRAF V600E mutated endometrial cancers, calling for prospective confirmatory clinical studies to be carried out in this setting.

## Abbreviations

BRAF: B-rapid accelerated fibrosarcoma
MEK: mitogen-activated protein/extracellular signal-regulated kinase kinase
MSI-H: microsatellite instability high
BRCA 1/2: breast cancer susceptibility protein ½
RECIST 1.1 criteria: response evaluation criteria in solid tumors criteria
